# A case of retroperitoneal dedifferentiated liposarcoma successfully treated by neoadjuvant chemotherapy and subsequent surgery

**DOI:** 10.1186/s40792-020-00865-2

**Published:** 2020-05-24

**Authors:** Yukihiro Yokoyama, Yoshihiro Nishida, Kunihiro Ikuta, Masato Nagino

**Affiliations:** 1 Department of Surgery, Nagoya University Graduate School of Medicine, 65 Tsurumai-cho, Showa-ku, Nagoya, Aichi 466-8550 Japan; 2 Department of Orthopedics, Nagoya University Graduate School of Medicine, 65 Tsurumai-cho, Showa-ku, Nagoya, Aichi 466-8550 Japan

**Keywords:** Retroperitoneal liposarcoma, Dedifferentiated, Chemotherapy, Complete pathological response

## Abstract

**Background:**

Retroperitoneal liposarcoma (RPLS) is the most commonly observed soft tissue sarcoma in the retroperitoneal space. Although the beneficial effect of chemotherapy for RPLS is controversial, there are some reports that have shown a considerable tumor-suppressive effect of chemotherapy in RPLS. We demonstrate a case of dedifferentiated RPLS, which was initially considered inoperable but was successfully treated by neoadjuvant chemotherapy and subsequent curative resection.

**Case presentation:**

A 59-year-old female was referred to our hospital with a chief complaint of right lower quadrant abdominal pain. Abdominal computed tomography revealed a large retroperitoneal tumor with a maximum diameter of 11 cm. The tumor involved retroperitoneal major vasculatures, such as the right common iliac vein and artery, as well as the right psoas muscle and femoral nerve. The right ureter was also involved and obstructed by the tumor. A biopsy was performed through the retroperitoneal route, and the tumor was diagnosed as a dedifferentiated liposarcoma with the Fédération Nationale des Centres de Lutte Contre le Cancer grade 3. Because the tumor was highly invasive and complete resection was not feasible, we decided to administer neoadjuvant chemotherapy with doxorubicin and ifosfamide (AI). After completing 6 courses of AI, the tumor size was considerably reduced, and we decided to perform surgery with curative intent. Before laparotomy, femoro-femoral arterial bypass was performed to prepare for the right common iliac artery resection. Thereafter, the patient underwent laparotomy and tumor resection combined with right nephrectomy, resection of the right common iliac artery and vein, and resection of the right psoas muscle and femoral nerve. The postoperative course was uneventful, although the patient needed a walking brace to support her gait. The pathological findings indicated a 99% disappearance of tumor cells. The patient was healthy without any complaints after 1 year of surgery, and a follow-up CT scan revealed no tumor recurrence.

**Conclusions:**

To the best of our knowledge, this is the first report that showed a nearly complete pathological response to AI in dedifferentiated RPLS, which was subsequently completely resected.

## Background

Although retroperitoneal sarcoma (RPS) is a rare entity, any general surgeon may have several opportunities to diagnose and treat this disease. Among various histological subtypes of RPS, retroperitoneal liposarcoma (RPLS) is the most commonly diagnosed tumor, accounting for approximately 30% of RPS [1].

Surgical resection is a standard procedure for RPLS, and radiotherapy [2, 3] or chemoradiotherapy [2–5] is occasionally combined as a neoadjuvant or adjuvant treatment. Chemotherapy and radiotherapy have been shown to be effective in soft tissue sarcoma in the extremities [5, 6]. However, the beneficial effects of these therapies for RPS are still controversial [2–4]. Nevertheless, there are some reports that have shown a substantial tumor-suppressive effect of chemotherapy or chemoradiotherapy for RPLS [7].

In this report, we demonstrate a case of dedifferentiated RPLS, which was initially considered an inoperable lesion but was successfully treated by neoadjuvant chemotherapy and subsequent curative resection.

## Case presentation

**Fig. 1:**
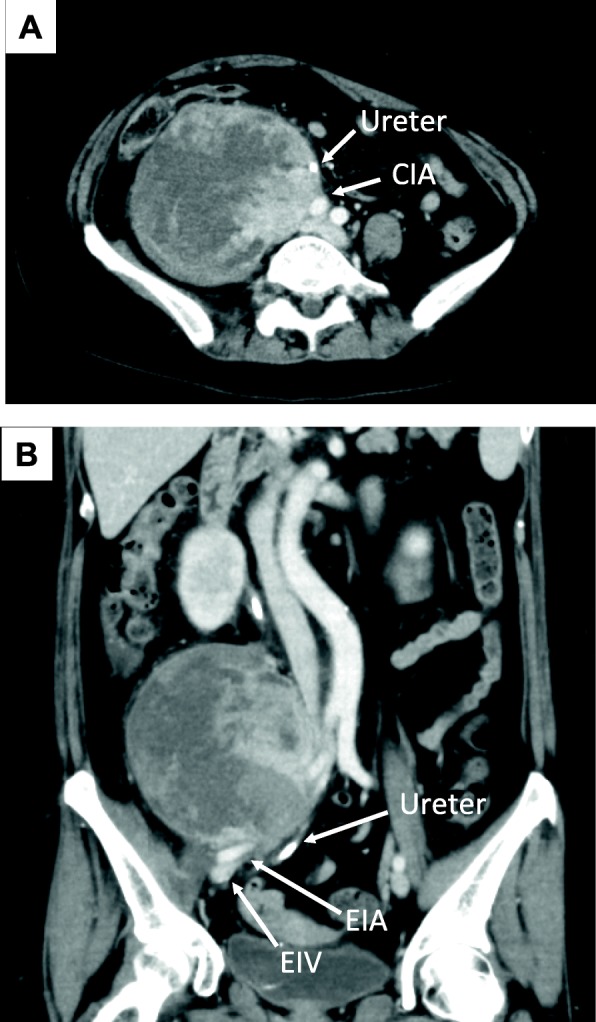
CT scan images of the retroperitoneal tumor. **a** Axial image. **b** Sagittal image after multiplanar reconstruction. CIA, common iliac artery; EIA, external iliac artery; EIV, external iliac vein

**Fig. 2:**
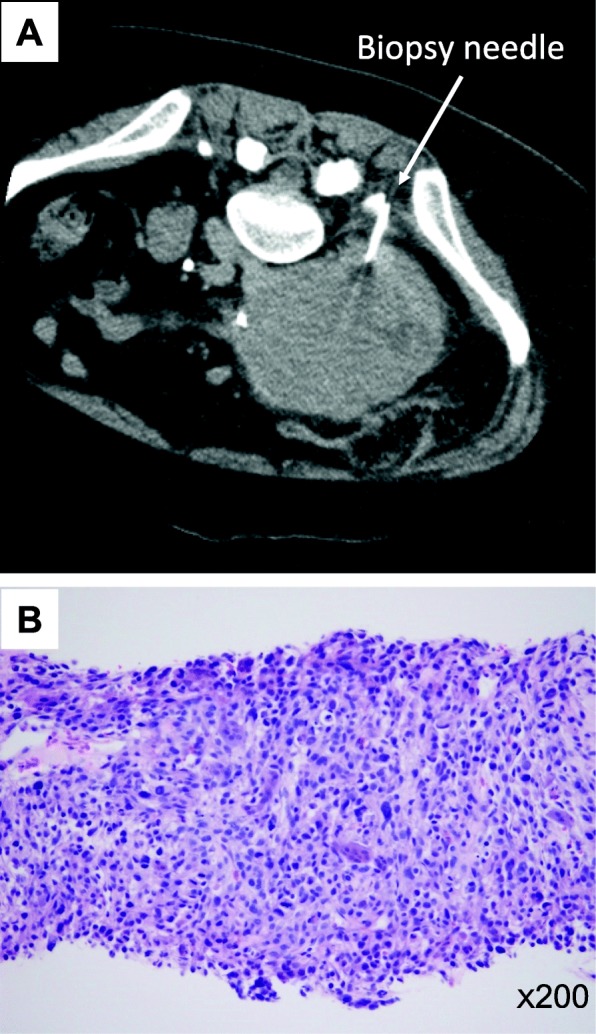
Biopsy of the tumor via a retroperitoneal approach. **a** CT scan during a biopsy. **b** Hematoxylin and eosin staining of the biopsy specimen

**Fig. 3:**
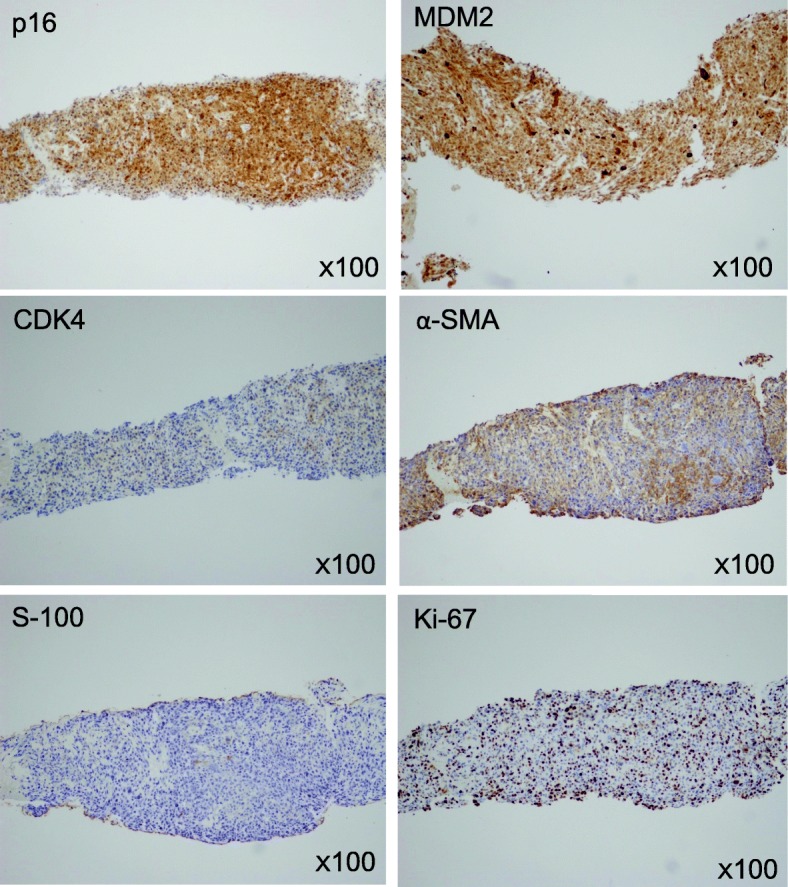
Immunohistochemistry of the biopsy specimen

**Fig. 4:**
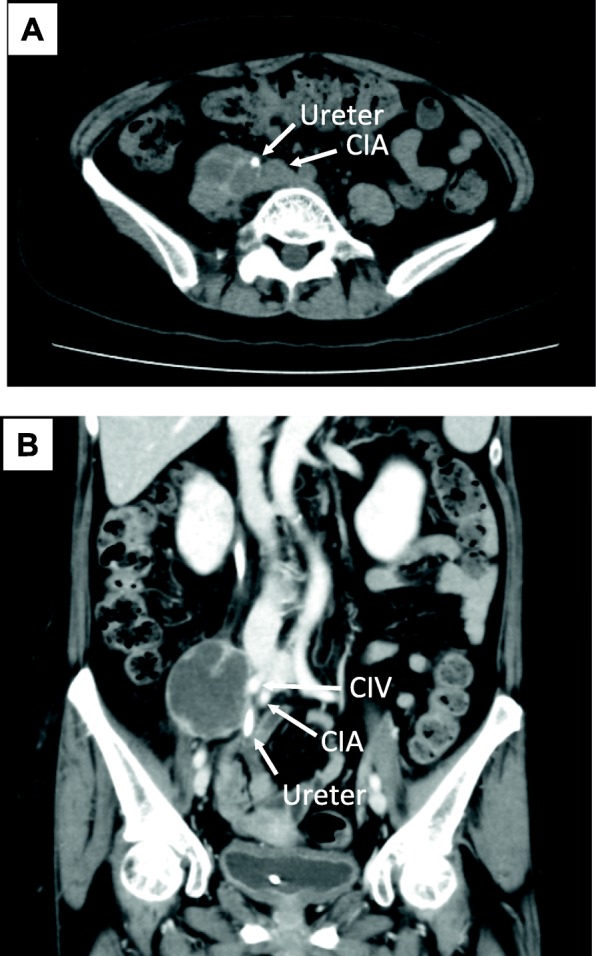
CT scans of retroperitoneal tumors after 6 courses of chemotherapy with AI. **a** Axial image. **b** Sagittal image after multiplanar reconstruction. CIA, common iliac artery; CIV, common iliac vein

A 59-year-old female was referred to our hospital with a chief complaint of right lower quadrant abdominal pain. An abdominal computed tomography (CT) scan revealed a multilobed retroperitoneal tumor with a maximum diameter of 11 cm (Fig. 1a, b). The tumor involved retroperitoneal major vasculatures, such as the right common iliac vein and artery, as well as the right psoas muscle and femoral nerve. The right ureter was also involved and obstructed by the tumor, and a ureteral stent was placed in another hospital for urinary drainage. A biopsy was performed through the retroperitoneal route for the histologic diagnosis (Fig. 2a). Hematoxylin and eosin staining of the biopsy specimens revealed pleomorphic tumor cells with scattered mitoses (Fig. 2b). Immunohistochemistry of the biopsy specimen was performed for the representative markers of liposarcoma (p16, MDM2, and CDK4), leiomyosarcoma (α-SMA), and neurogenic tumor (S-100). The results of immunohistochemistry revealed positive staining for p16, MDM2, and α-SMA and negative staining for CDK4 and S-100 (Fig. 3). The Ki-67 index was approximately 40%. Although the tumor partially had a character of leiomyosarcoma, the lesion was ultimately diagnosed as dedifferentiated liposarcoma with the Fédération Nationale des Centres de Lutte Contre le Cancer (FNCLCC) grade 3. Because the tumor was highly invasive and complete resection was not feasible, we decided to administer neoadjuvant chemotherapy with doxorubicin and ifosfamide (AI). The patient received six courses of doxorubicin (20 mg/m^2^ per day on days 1–3) and ifosfamide (1.6 g/m^2^ per day on days 1–5). The treatment was repeated every 3 weeks until the fifth course. In the sixth course, the treatment was delayed 1 week because of thrombocytopenia. After completing 6 courses of AI, the tumor size was considerably reduced (Fig. 4a, b), and we decided to perform surgery with curative intent. The surgery was performed 6 months after the first diagnosis.

**Fig. 5:**
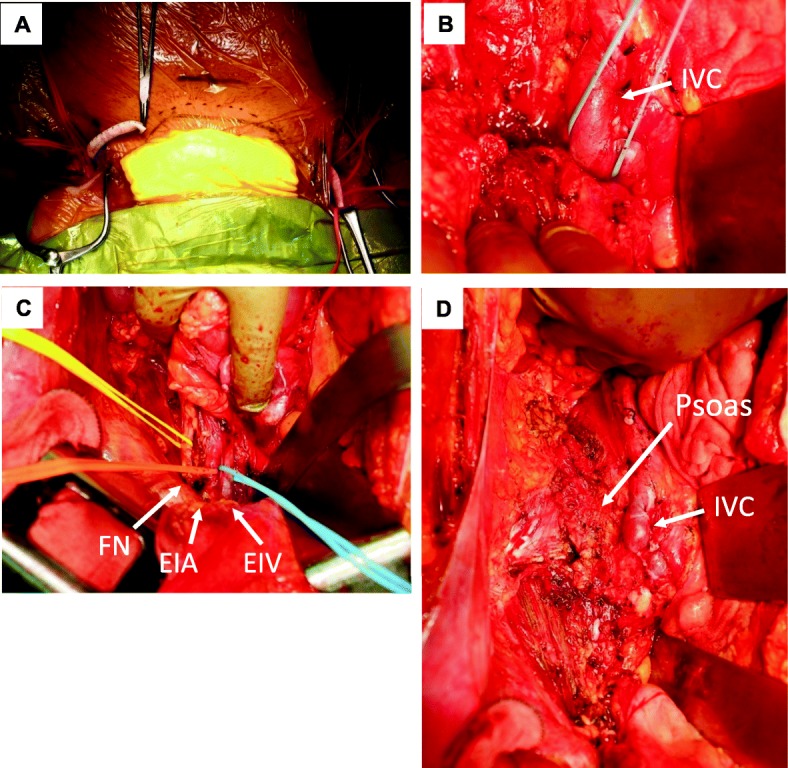
Intraoperative photos. **a** Construction of the femoro-femoral bypass using a 10-mm artificial graft. **b** Before resecting the common iliac vein. IVC, inferior vena cava. **c** Before resecting the femoral nerve (FN), external iliac artery (EIA), and external iliac vein (EIV). **d** After tumor resection

Before laparotomy, femoro-femoral arterial bypass was performed to prepare for the right common iliac artery resection (Fig. 5a). Thereafter, the patient underwent laparotomy and tumor resection combined with right nephrectomy, resection of the right common iliac artery and vein, and resection of the right psoas muscle and femoral nerve (Fig. 5b–d). The right common iliac vein was not reconstructed. The postoperative course was uneventful, although the patient needed a walking brace to support her gait.

**Fig. 6:**
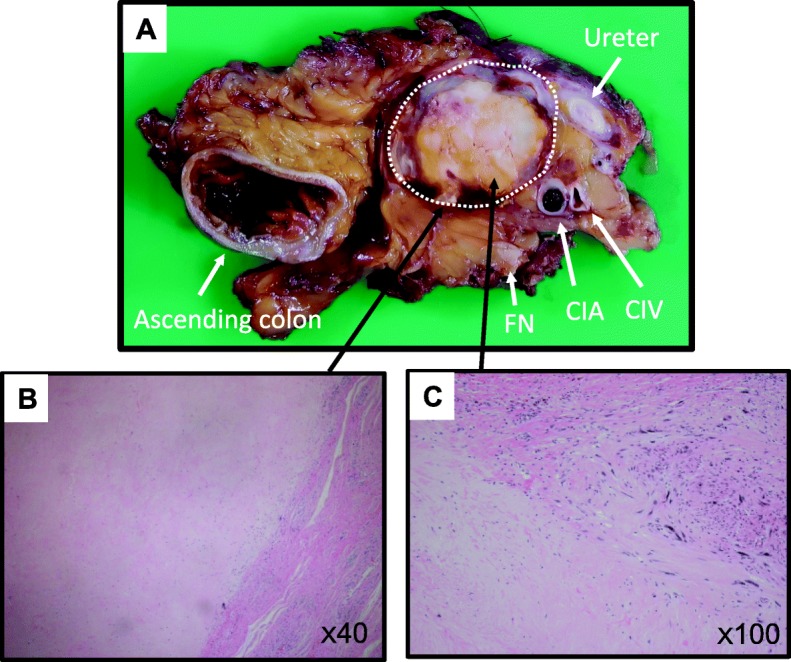
Pathological findings. **a** Photograph of the resected specimen (transected at the maximum diameter of the tumor, which is encircled by the dotted line). **b** Hematoxylin and eosin staining of the resected specimen with low-power field. **c** High-power field showing a small number of atypical cells

Macroscopically, the resected specimen revealed a whitish solid tumor surrounded by fibrosis (Fig. 6a). The pathological examination indicated a 99% disappearance of tumor cells (Fig. 6b), and only a small number of atypical cells were partially observed (Fig. 6c). Although the Ki-67 index was much lower (< 1%) in these cells, other immunohistochemical characteristics were similar to those of the biopsy specimen. The surgical margin was negative for tumor cells. The patient was healthy without any complaints after 1 year of surgery, and a follow-up CT scan revealed no tumor recurrence.

## Discussion

Retroperitoneal tumor (RPT) is a tumor originating in the retroperitoneal space compartmentalized by the parietal peritoneum and transversalis fascia [8]. Because of its anatomical position, RPT does not have accompanying clinical symptoms unless it becomes a large tumor. Therefore, when RPT is diagnosed, it usually appears as a large tumor. Approximately 90% of RPTs are categorized as mesodermal tumors, and liposarcoma is the most common pathological subtype in RPS. RPLS is divided into the well-differentiated, dedifferentiated, myxoid/round, and pleomorphic types according to histological features. Although the prognosis of well-differentiated liposarcoma is relatively favorable, that of other pathological types of liposarcoma is poor, especially that of dedifferentiated subtype [9]. In general, well-differentiated RPLS may not infiltrate other organs or major intra-abdominal structures, whereas dedifferentiated RPLS frequently infiltrates and involves surrounding tissues.

Surgical resection is the only curative therapy for RPLS. However, chemotherapy, radiotherapy, or chemoradiotherapy is sometimes combined with surgical resection as an option of adjuvant therapy [3, 4]. Several reports have indicated that neoadjuvant radiotherapy or chemoradiotherapy for RPLS is beneficial in patients undergoing curative resection for RPLS [2, 3]. However, the most recent report from the Tranasatlantic Retroperitoneal Sarcoma Working Group analyzing 607 patients with localized RPLS showed controversial results [10]. Although perioperative radiotherapy was associated with better local control in univariable unadjusted analysis, this significant benefit was lost after the inverse probability of treatment weighting using propensity scores. Therefore, the appropriate selection of radiotherapy remains challenging and prospective randomized trial is required.

Other than radiotherapy, there are few effective drugs with high evidence levels for RPLS. The response rate to doxorubicin, ifosfamide, trabectedin, eribulin, pazopanib, and some other antitumor agents has been shown to be approximately 20% [11–13]. Unfortunately, no RCT has investigated the effectiveness of neoadjuvant chemotherapy for RPLS. Nevertheless, there are some case reports that have indicated a significant effectiveness of chemotherapy [7, 14–18]. For instance, Kus et al. reported a complete response of a recurrent-metastatic RPLS [7]. These results indicated that there are some RPLSs that are highly sensitive to antitumor drugs. This study showed a case of dedifferentiated liposarcoma that revealed an extreme tumor-suppressive effect of AI chemotherapy. To the best of our knowledge, this is the only report to show a nearly complete pathological response to AI in dedifferentiated RPLS, which was successfully resected by subsequent aggressive surgery. It should be noted, however, that the reason why the current case showed such a favorable response is completely unknown. A recent report showed that the expression level of particular microRNAs in soft tissue sarcoma tissue samples was different between non-responders and responders to chemotherapy [19]. Further molecular and genetic investigations are necessary to elucidate the mechanism that regulates sensitivity to chemotherapy in RPLS.

## Conclusions

We present a case of dedifferentiated RPLS that showed a high sensitivity to AI treatment and was successfully treated by subsequent curative surgery.

## Data Availability

We would not like to share data other than those described in the paper because they include personal information.

## Abbreviations

CT: Computed tomography
RCT: Randomized controlled trial
RPLS: Retroperitoneal liposarcoma
RPT: Retroperitoneal tumor
